# Salivary Alterations in Autoimmune Thyroid Diseases: A Systematic Review

**DOI:** 10.3390/ijerph20064849

**Published:** 2023-03-09

**Authors:** Martyna Ortarzewska, Kacper Nijakowski, Julia Kolasińska, Dawid Gruszczyński, Marek A. Ruchała, Anna Lehmann, Anna Surdacka

**Affiliations:** 1 Department of Conservative Dentistry and Endodontics, Poznan University of Medical Sciences, 60-812 Poznan, Poland; 2 Student’s Scientific Group in Department of Conservative Dentistry and Endodontics, Poznan University of Medical Sciences, 60-812 Poznan, Poland

**Keywords:** autoimmune thyroid disease, Graves’ disease, Hashimoto’s thyroiditis, saliva, biomarkers, salivation

## Abstract

Autoimmune thyroid disease (AITD) is a dysregulation of the immune system that causes an attack on the thyroid gland. Two major clinical manifestations are Hashimoto’s thyroiditis and Graves’ disease. Saliva performs many functions and, importantly, has the potential for easy, non-invasive diagnostics of several systemic disorders. This systematic review was designed to answer the question whether salivary alterations are reliable for the diagnosis of autoimmune thyroid diseases. Following the inclusion and exclusion criteria, fifteen studies were included. Due to their heterogeneity, saliva analysis was divided into two subgroups: quantitative assessment analysing salivation and qualitative assessment concerning potential salivary biomarkers for AITD. In addition to detecting altered levels of thyroid hormones and antibodies, salivary changes were also observed in the concentrations of total protein, cytokines and chemokines, as well as markers of oxidative status. According to the saliva flow rate values, significantly reduced saliva secretion was observed in patients with HT. In conclusion, it is not possible to unequivocally state if salivary biomarkers can potentially be used in autoimmune thyroid disease diagnosis. Therefore, further investigations, including salivation disorders, are necessary to validate these findings.

## 1. Introduction

Autoimmune thyroid disease (AITD) is a dysregulation of the immune system that results in an attack on the thyroid gland. Hashimoto’s thyroiditis (HT) and Graves’ disease (GD) are its two major clinical manifestations [1]. It is estimated that HT and GD affect about 5% of the general population. This evaluation indicates that they are the most common autoimmune disorder affecting humans [2]. AITD arises due to the loss of tolerance to thyroid antigens in genetically liable individuals in association with environmental factors, such as microbiota alterations [3,4,5]. Both manifestations are characterised by infiltration of the thyroid by T and B cells, reactive to thyroid antigens, the production of thyroid autoantibodies and abnormal thyroid functions [6]. The major autoantigens in AITD are thyroid peroxidase (TPO), thyroglobulin (Tg) and thyroid-stimulating hormone receptor (TSHR) [7]. Autoimmune thyroid disease is more common among women than men [8]. Referring to HT, the inflammation leads to follicular cell damage and thyroid gland destruction and, in consequence, the development of hypothyroidism. On the other hand, in GD the presence of thyroid-stimulating antibodies, which activate the thyrotropin receptor (TSHR) on thyrocytes, leads to thyroid hyperplasia and, finally, hyperthyroidism [1,3,6]. Due to various effects on thyroid function, HT and GD present different clinical symptoms. HT is associated with changeable and not specific features, such as fatigue, weakness, nervousness, irritability, anaemia and skin problems [9,10], whereas GD manifests in tremors, heat sensitivity, weight loss, anxiety, enlargement of the thyroid gland (goitre) or alterations in menstrual cycles [11,12].

Saliva is an oral fluid containing the mixture of major and minor salivary gland secretions, gingival crevicular fluid, cellular debris and microorganisms [13]. Saliva performs many varied functions, such as protection of the oral cavity, digestion due to salivary amylase, defence against microorganisms and, importantly, diagnostic functions [14]. Saliva collection is easy, non-invasive and inexpensive, so its use in diagnostic tests is constantly growing [15]. In saliva, it is possible to find enzymes, hormones, antibodies, antimicrobial constituents, as well as growth factors, which enter from the blood by passing through the spaces between cells. The development of new and highly sensitive techniques (e.g., molecular diagnostics, nanotechnology) resulted in fewer limitations caused by a low concentration of constituents compared with the blood and more clinically practical use [16]. Oncological, endocrine, cardiovascular, rheumatic, autoimmune, neurological or infectious diseases are just some of the disorders in which saliva has impressive diagnostic potential [17].

Many studies prove that systemic autoimmune disorders can be accompanied by salivary gland dysfunction, which manifests in hyposalivation, like systemic lupus erythematosus, polymyositis, dermatomyositis, systemic scleroderma, mixed connective tissue disease [18] as well as Sjögren’s syndrome or rheumatoid arthritis [19]. Not only quantitative assessment of salivation but also qualitative assessment concerning biomarkers, like salivary interleukins, immunoglobulins, cytokines, hormones, enzymes and multiple other components is diagnostically useful [20].

There are signs that autoimmune thyroid diseases, like HT and GD, can also be accompanied by salivary gland dysfunction, and markers detected in saliva can have potential diagnostic value. Therefore, our systematic review was designed to answer the question “Are salivary alterations reliable for the diagnosis of autoimmune thyroid diseases?”, formulated according to the PICO (“Population”, “Intervention”, “Comparison”, “Outcome”).

## 2. Materials and Methods

### 2.1. Search Strategy and Data Extraction

A systematic review was conducted up to 27th September 2022, according to the Preferred Reporting Items for Systematic Reviews and Meta-Analyses (PRISMA) statement guidelines [21], using the databases PubMed, Scopus and Web of Science. The search formulas included:

- For PubMed: saliva* AND (((thyroid OR Graves) AND (orbitopathy OR ophthalmopathy)) OR ((Graves OR Hashimoto) AND (disease OR thyroiditis)));
- For Scopus: TITLE-ABS-KEY(saliva* AND (((thyroid OR Graves) AND (orbitopathy OR ophthalmopathy)) OR ((Graves OR Hashimoto) AND (disease OR thyroiditis))));
- For Web of Science: TS = (saliva* AND (((thyroid OR Graves) AND (orbitopathy OR ophthalmopathy)) OR ((Graves OR Hashimoto) AND (disease OR thyroiditis)))).

Records were screened by the title, abstract and full text by two independent investigators. Studies included in this review matched all the predefined criteria according to PICOS (“Population”, “Intervention”, “Comparison”, “Outcomes”, and “Study design”), as shown in Table 1. In general, the original studies on AITD patients (GD and HT) were selected for salivary alterations assessment—quantitative (e.g., hyposalivation) and/or qualitative (e.g., biochemical profile changes). A detailed search flowchart is presented in Section 3. The study protocol was registered in the international prospective register of systematic reviews PROSPERO (CRD42022370454).

### 2.2. Quality Assessment and Critical Appraisal for the Systematic Review of Included Studies

**Figure 1:**
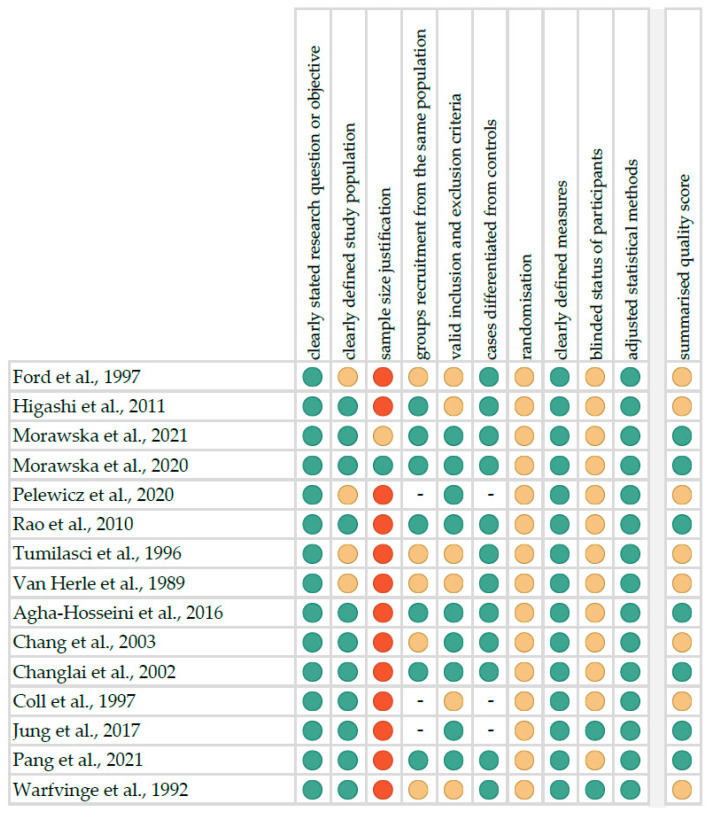
Quality assessment, including the main potential risk of bias (risk level: green—low, yellow—unspecified, red—high; quality score: green—good, yellow—intermediate, red—poor) [24,25,26,27,28,29,30,31,32,33,34,35,36,37,38].

The risk of bias in each individual study was assessed according to the “Study Quality Assessment Tool” issued by the National Heart, Lung and Blood Institute within the National Institute of Health [22]. These questionnaires were answered by two independent investigators, and any disagreements were resolved by discussion between them. The summarised quality assessment for every single study is reported in Figure 1. The most frequently encountered risks of bias were the absence of data regarding randomisation (all studies), blinding (thirteen studies) and sample size justification (thirteen studies). Critical appraisal was summarised by adding up the points for each criterion of potential risk (points: 1—low, 0.5—unspecified, 0—high). Seven studies (46.7%) were classified as having “good” quality (≥80% total score) and eight (53.3%) as “intermediate” (≥60% total score).

The level of evidence was assessed using the classification of the Oxford Centre for Evidence-Based Medicine levels for diagnosis [23]. All of the included studies have the third or fourth level of evidence (in this 5-graded scale).

## 3. Results

**Figure 2:**
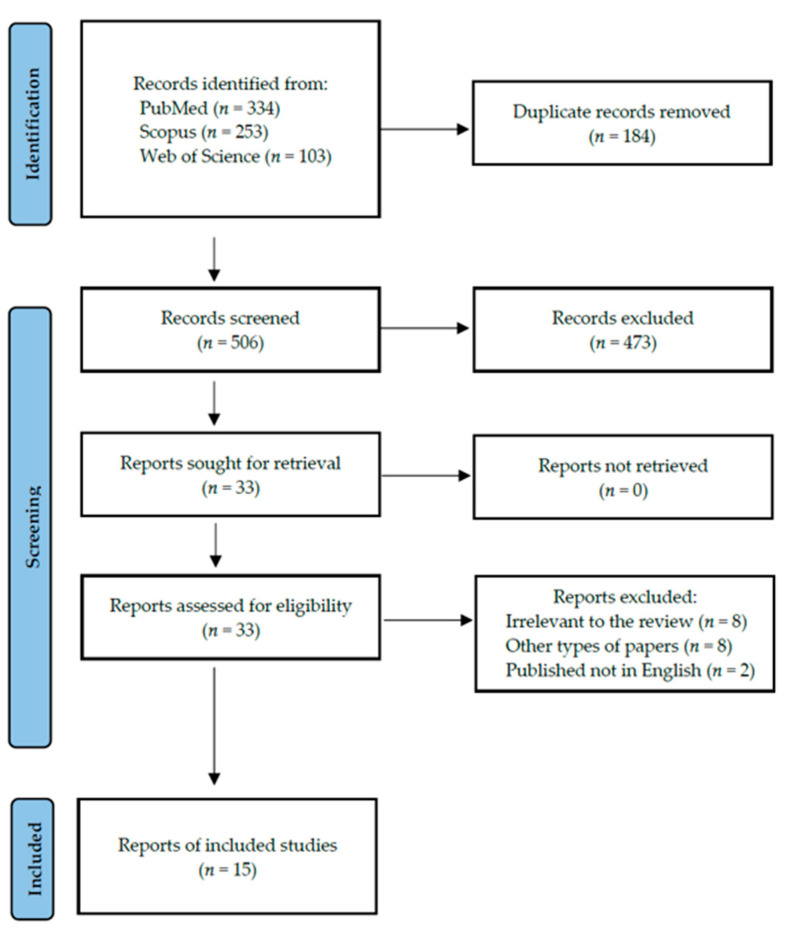
PRISMA flow diagram presenting the search strategy.

Following the search criteria, our systematic review included fifteen studies, demonstrating data collected in twelve different countries from a total of 473 participants with diagnosed autoimmune thyroid diseases (including 46 with GD and 427 with HT). Figure 2 shows the detailed selection strategy of the articles. The inclusion and exclusion criteria are presented in Section 2.

From each eligible study included in the present systematic review, we collected data about its general characteristics, such as year of publication and setting, involved participants, AITD diagnosis, inclusion and exclusion criteria of patients, smoking status and pharmacological treatment. Table 2 presents the general characteristics for studies dealing mainly with qualitative assessments of saliva. The detailed characteristics considering types of saliva, methods of collection, centrifugation, storing and laboratory analysis, method of salivation assessment, as well as potential salivary markers for AITD from these studies are reported in Table 3. The included studies similarly concerned both GD and HT patients, unfortunately rarely reporting smoking status. Not all studies had a control group due to the cross-sectional design. Two papers by the same authors present a similar group of patients with HT—however, one includes only untreated patients, and the other already treated, and in these studies, completely different markers were determined. Most studies concerned unstimulated saliva, and only a few—additionally stimulated. Only half of these studies determined flow rate. Each study identified different types of markers (e.g., cytokines, chemokines, antioxidants, hormones) without being able to compare these results. Centrifuge and storage parameters varied, but freezing temperatures were typically below −20 °C until the biochemical analyses. Moreover, Table 4 shows the general characteristics for studies describing only a quantitative assessment of saliva. Most of these studies involved patients with HT. Almost all evaluated both types of saliva (unstimulated and stimulated) with a scintigraphic evaluation of salivary gland functions.

Table 5 demonstrates the reported saliva flow rate values. Significantly reduced saliva secretion can be observed in patients with HT, especially for unstimulated saliva.

## 4. Discussion

In the following discussion, saliva analysis was divided into two subgroups: quantitative assessment analysing salivation and qualitative assessment concerning markers. This section structure was established intentionally because of the considerable heterogeneity of the discussed studies.

### 4.1. Quantitative Assessment of Saliva

For many years, researchers have been using sialometry and scintigraphy in the process of quantitative analysis of saliva, which assesses salivary gland function. Many scientists see the potential of these methods in the study of thyroid diseases (TD).

In 1992, Warfvinge et al. [38] was one of the first to conduct a study investigating salivary gland involvement in autoimmune thyroiditis (AT). Salivary gland function was assessed by unstimulated whole sialometry, lower lip salivary gland biopsy and salivary gland scintigraphy. Eleven of 19 cases of Hashimoto’s thyroiditis (HT) showed different degrees of salivary gland implication. In addition, the frequency of primary Sjögren’s syndrome (SS) among patients with autoimmune thyroiditis was established with lacrimal gland function tests—six of these cases fulfilled the criteria. The authors suggested that meaningful involvement of salivary glands may occur. Such findings indicate the concept that focal lymphocytic infiltration of minor salivary glands may represent an epiphenomenon, somehow related to a common tendency of the immunoreactive cells of diseases such as primary SS and AT to interact with epithelial tissue.

A decade later, the team of Changlai et al. [34] also investigated salivary function in patients with autoimmune thyroiditis, but in a group of 40 individuals. Among the exclusion criteria, apart from smoking, were bad blood sugar control, autonomic neuropathy, immunorheumatic and other endocrine, gastrointestinal, hepatobiliary and renal diseases. Participants were divided into two subgroups: patients with xerostomia and patients without xerostomia, and then salivary function was evaluated by objective, quantitative salivary scintigraphy. Uptake and excretion ratios were calculated. The study showed impaired salivary function in patients with AT. Significantly poorer salivary function was found in Hashimoto’s thyroiditis participants with xerostomia.

A year later, Chang et al. [33] published a quite similar study considering salivary gland function in patients with autoimmune thyroiditis, but carried out on a wider group—120 participants. The same exclusion criteria and division into subgroups were used. Interestingly, the results and conclusions after objective quantitative salivary scintigraphy were similar. The authors found significantly decreased uptake and excretion ratios values in AT patients with xerostomia which proved impaired salivary function.

Moreover, Agha-Hosseini et al. [32] focused their research on salivation in Hashimoto’s thyroiditis. Forty non-smoker women diagnosed with autoimmune thyroiditis without any other systemic disease were enrolled in the study. Stimulated and unstimulated whole saliva samples were collected, and the flow rate was calculated in millilitres per minute. Patients with HT demonstrated higher xerostomia, especially the difference in unstimulated salivary flow rate was significant. The authors suspected that autoimmune diseases could be accompanied by salivary gland dysfunction because of cytokines in the autoimmune process or thyroid hormone disorder.

In the year 2021, Pang et al. [37] conducted a study investigating salivary gland function in women with Hashimoto’s thyroiditis accompanied by differentiated thyroid cancer, interestingly without xerostomia. Moreover, for the first time, they analysed the correlation with auto-thyroid antibodies. The authors concluded that women without xerostomia may not present salivary functional impairment during hypothyroidism, despite Hashimoto’s thyroiditis. Furthermore, the serum thyroid autoantibody and TSH values correlated with salivary excretive function.

Among thyroid diseases, not only Hashimoto’s thyroiditis is the subject of research. The goal of the study led by Coll et al. [35] was to evaluate the prevalence of subclinical Sjögren’s syndrome features among individuals with thyroid diseases, except HT, also Graves’ disease and primary myxedema. Salivary scintigraphy, labial salivary gland biopsy and immunohistopathological studies were carried out on 176 participants. The results presented that a third of patients with autoimmune TD had Sjögren’s syndrome features. The authors postulated that these diseases are associated and closely related pathogenetically.

Jung et al. [36] also examined a large group, mainly women, to estimate the prevalence of thyroid disease, like Hashimoto’s thyroiditis, subacute thyroiditis, subclinical hypothyroidism and Graves’ disease in participants with xerostomia. Furthermore, they were concerned about the efficacy of salivary gland scintigraphy in the detection of TD. Based on results showing that more than half of the patients with xerostomia suffered from thyroid diseases, the researchers suggested that symptoms of xerostomia should be considered as possible signs of them.

To sum up, patients with HT demonstrate reduced saliva secretion. This is true since around half of these patients have clinical and histopathological features of salivary gland inflammation, and a substantial proportion of them fulfil the criteria for Sjogren’s syndrome.

### 4.2. Qualitative Assessment of Saliva

Additionally, qualitative assessment of saliva, that is, the detection of biomarkers, is used in research related to thyroid diseases.

Morawska et al. [27] juxtaposed stimulated and unstimulated whole saliva of Hashimoto’s thyroiditis patients, considering oxidation markers. HT individuals presented significantly lower levels of salivary amylase activity, reduced glutathione and uric acid compared to those in the control group. However, the activity of peroxidase, catalase as well as levels of total protein and IL-1 were notably higher in the examined subjects. Generally, Hashimoto’s thyroiditis patients showed lower antioxidant potential. A significant increase in oxidatively modified molecules in saliva suggests the failure of the salivary gland antioxidant barrier to combat excess ROS production. In these patients, salivary oxidative stress appeared to be closely connected with autoimmunity-related inflammation, and not with the level of thyroid hormones or TSH. Moreover, the decreased secretory function of the submandibular glands of HT female patients in euthyreosis manifested with a significant reduction of unstimulated saliva secretion.

A year later, a similar research team [26] investigated cytokines, chemokines and growth factor levels in patients with Hashimoto’s thyroiditis in unstimulated whole saliva. Endocrine individuals demonstrated elevated levels of cytokines associated with Th1 lymphocyte activation, interleukins connected with Th2 lymphocyte activation, TNF-α, IL-12 (p40), HGF, IL-1α, IL-1β, IL-1RA, CCL27/CTACK and CXCL1/Gro-α, G-CSF and VEGF. Additionally, the general protein concentration was higher than that in the healthy group. On the contrary, levels of salivary IL-8 and IL-10 were lower. The highest predictive values showed IFN-γ and IL-12 (AUC = 0.910, 95% CI: 0.828–0.993 and AUC = 0.861, 95% CI: 0.755–0.966, respectively). The authors concluded that the cytokine levels in the saliva and plasma of patients with untreated euthyroid HT did not indicate the dominance of any of the branches of the immune response. However, salivary IL-12 (p40) may be helpful in assessing the progression of autoimmunity-related inflammation in the course of HT. Furthermore, IL-6 and IL-1 as well as INF-γ, TNF-α, and IL-12 may be potential biomarkers for salivary gland dysfunction in HT.

On the other hand, Rao et al. [29] examined C-reactive protein (CRP) levels in patients with Hashimoto’s thyroiditis, but also subacute thyroiditis (ST). They determined whether there were differences compared to the control group. Saliva collected from HT patients did not show significant CRP concentration disproportions. However, ST presented higher levels of CRP, as opposed to HT and euthyroid groups. It indicated that salivary CRP could be used as an inflammatory marker, which could also be helpful with clinical evaluation by using a non-invasive and minimally stressful sampling methodology.

Tumilasci et al. [30] considered thyrotropin receptor antibodies (TRAb) in parotid saliva and serum in patients with Graves’ disease, Hashitoxicosis and Hashimoto’s thyroiditis. They compared levels of TRAb in saliva and serum. It appeared they were higher in stimulated saliva rather than in serum in all three aforementioned diseases. The differences between the pathologies in serum and saliva were very slight in considered ailments. The significant one was between Graves’ and Hashitoxicosis individuals. Serum TRAb may be used to diagnose and evaluate Graves’ progression. The authors recommended the parotid salivary assay as a complementary study in patients with thyroid diseases due to a good correlation between salivary and serum TRAb levels.

Similarly, Van Herle et al. [31] were interested not only in HT but also in other various thyroid disorders. They studied the appearance of serum TgAb in saliva of patients who had TgAb in their sera. There were no statistically significant differences between the control and thyroid-malfunction groups for mean thyroglobulin salivary concentration. Individuals with elevated Tg levels and TgAb negative sera did not display higher salivary Tg extents. In spite of detecting thyroglobulin in saliva, it was impossible to use this as a marker to assess the risk of thyroid cancer or Graves’ disease repercussions.

Some scientists focused their studies on hyperthyroidism, especially Graves’ disease. Ford et al. [24] investigated possible changes in saliva constituents in hyperthyroidism after administering an ablative dose of radioactive iodine. The examined group presented an increase in salivary flow, as well as urate and potassium concentrations. On the other hand, they observed a decrease in total protein and calcium concentrations along with lactate dehydrogenase activity. After the intake of radioactive 370 MBq iodine, there were no significant changes for most of the salivary components. The researchers observed some positive trends in the levels of N-acetylglucosaminidase (NAG), IgA and total protein. It could be attributed to the effects of radiation on salivary glands, thyroid condition alteration or both. Although the team detected some changes, concentrations of different components varied based on the size of the subgroups. The authors suggested that clinically significant salivary gland dysfunction was associated with the minor changes observed in total protein, NAG activity and IgA.

Patients suffering from Graves’ disease were also enrolled in a study conducted by Higashi et al. [25]. The researchers examined thyroxine levels in saliva. In order to obtain the results, they used a stable isotope-dilution liquid chromatography/tandem mass spectrometry method. The results showed that T4 concentrations were considerably higher in studied subjects, as opposed to that in healthy individuals. It should be emphasised that the measurements of salivary T4 could serve as a potential help in diagnosing Graves’ disease because of their repetitiveness and simplicity to perform.

Furthermore, Pelewicz et al. [28] examined patients with orbitopathy, which is a consequence of Graves’ disease. The authors decided to check the impact of intravenous methylprednisolone (IVMP) treatment in a group that suffered from Graves’ orbitopathy. They noted no significant decrease in salivary cortisol before the administration of the 12th IVMP pulse. Additionally, the researchers examined patients with oral prednisone therapy and came to the conclusion that there was a statistically significant decrease in salivary cortisol levels. However, they did not note a considerable difference among age groups.

### 4.3. Study Limitations

Among the limitations of the review, the limitations of the individual included studies should be pointed out first and foremost. Some studies had relatively small sample sizes, without any justification, and not rarely unmatched groups by age or gender. Only one research team tried to carry out sample size justification. Both randomisation and blinding methods have not been described, but they do not significantly affect the performed studies. A few articles (especially older ones) did not accurately present the inclusion and exclusion criteria for participants in the study. The majority of participants suffered from HT, and a significant minority from GD. 

Moreover, the measurement of biomarkers in saliva has a lot of parameters that must be considered (oral diseases, such as gingivitis, smoking status, saliva volume, environmental temperature, season of the year as well as diurnal fluctuations of saliva production). Not all of the included studies considered these parameters in their analyses. Additionally, the appropriate method of storing and processing the specimens was not always well described for the different substances used as biomarkers.

Diverse research models reporting different types of data did not allow to perform any meta-analyses. In terms of diagnostic reliability, only one of the studies presented AUC values (outside the article in the supplementary materials). In general, it must be said that there is a lack of reliable reports on the effect of AITD on the composition of secreted saliva, as well as its flow rate.

## 5. Conclusions

Based on the systematic review, it is not possible to unequivocally conclude if salivary biomarkers can potentially be used in autoimmune thyroid disease diagnosis. Previous studies show numerous limitations, which underlines the lack of scientific evidence in this area about the salivary diagnostics of AITD. So far, AITD diagnosis has been relatively easily performed on the basis of clinical and laboratory tests of thyroid function as well as the presence of thyroid-disease-specific autoantibodies. Therefore, further investigations, including salivation disorders, are necessary to validate these findings.

## Figures and Tables

**Table 1:** Inclusion and exclusion criteria according to PICOS.

Parameter	Inclusion Criteria	Exclusion Criteria
Population	Patients with autoimmune thyroid diseases, including Graves’ disease and Hashimoto’s thyroiditis—aged from 0 to 99 years, both genders	Patients with other autoimmune diseases
Intervention	Not applicable	
Comparison	Not applicable	
Outcomes	Salivary alterations quantitative (e.g., hyposalivation) and/or qualitative (e.g., biomolecules)	Other salivary alterations (e.g., microbiota)
Study design	Case-control, cohort and cross-sectional studies	Literature reviews, case reports, expert opinion, letters to the editor, conference reports
	Published until 27th September 2022	Not published in English

**Table 2:** General characteristics of included studies considering mainly qualitative assessments of saliva.

Author, Year	Setting	Study Group (F/M); Age	Control Group (F/M); Age	AITD Diagnosis	Inclusion Criteria	Exclusion Criteria	Smoking Status	Pharmacological Treatment
Ford et al., 1997 [24]	New Zealand	38 (NR); NR	93 (NR); NR	GD	Hyperthyroidism	NR	NR	CBZ, 370 MBq 131I
Higashi et al., 2011 [25]	Japan	2 (2/0); 22–43	16 (6/10); 22–41	GD	Diagnosed with GD	NR	NR	Untreated
Morawska et al., 2021 [26]	Poland	25 (25/0); 34.5 (27.8–41.5)	25 (25/0); 34.3 (27.2–42.0)	HT	Euthyroid HT who had never been treated with synthetic or natural thyroid hormones or had any other treatments applied, no other diseases	BMI < 18.5 and >25, periodontal disease, candidiasis, inflammation in the oral mucosa, poor oral hygiene, presence of multiple dental deposits, medications on a permanent basis, other than second phase of the menstrual cycle (between the 18th and 25th day), weight-loss diet and significantly changed lifestyle (during 6 months preceding the research), consumption of alcohol not only occasionally and addiction to other stimulants	Non-smokers	Untreated
Morawska et al., 2020 [27]	Poland	45 (45/0); 35 (29–43)	45 (45/0); 35 (29–43)	HT	Not any associated diseases, including other autoimmune diseases or depression; Ctrl: normal serum TSH, fT4, anti-TG and anti-TPO levels as well as thyroid imaging (homogenous parenchyma without nodules) on USG	BMI < 18.5 and >25, any drugs that could affect saliva secretion (mainly antidepressants or drugs for hypertension) or its redox status (vitamins, antioxidants) within 3 months prior to saliva collection, reducing diet, periodontitis, gingivitis, active foci of odontogenic infections, any amount of alcohol or other stimulants	Non-smokers	24 patients treated with LT4 (doses from 50 to 150 mg; the last tablet taken 24 h before the hormone level test) and 21 patients untreated
Pelewicz et al., 2020 [28]	Poland	14 (11/3); NR	NA	GO	Euthyroidism within the last 3 months before the study	Diagnosis of adrenal insufficiency, treatment with GCs or medication altering the plasma CBG and serum DHEA-S levels within the last 6 months before the study, medical conditions altering CBG levels	NR	IVMP followed by oral prednisone
Rao et al., 2010 [29]	India	30 (28/2); 28.85 ± 8.83	20 (17/3); 31.82 ± 9.39	HT	Clinical features of hypothyroidism	Ctrl: existence of any comorbid cardiac, autoimmune, infectious, musculoskeletal, malignant disease, oral disease; recent history of operation or trauma; pregnancy, peri- or postmenopausal age; drug regimen	NR	NR
Tumilasci et al., 1996 [30]	Argentina	GD: 8 (NR); NR HT: 10 (NR); NR	6 (NR); NR	GD, HT	Diagnosed with AITD	NR	NR	LT4
Van Herle et al., 1989 [31]	USA	GD: 21 (NR); NR HT: 9 (NR); NR	10 (NR); NR	GD, HT	Ctrl: no thyroid disorders; age between 14 and 52 years	NR	NR	NR

Legend: F, females; M, males; AITD, autoimmune thyroid disease; GD, Graves’ disease; GO, Graves’ orbitopathy; HT, Hashimoto’s thyroiditis; Ctrl, controls; NR, not reported; NA, not applicable; BMI, body mass index; CBZ, carbimazole; TSH, thyroid stimulating hormone; fT4, free thyroxine; anti-TG, thyroglobulin antibody; anti-TPO, thyroid peroxidase antibody; USG, ultrasonography; LT4, levothyroxine; GCs, glucocorticoids; CBG, cortisol-binding globulin; DHEA-S, dehydroepiandrosterone sulphate; IVMP, intravenous methylprednisolone.

**Table 3:** Detailed characteristics of included studies considering mainly qualitative assessments of saliva.

Author, Year	AITD Diagnosis	Type of Saliva and Method of Collection	Centrifugation and Storing	Method of Analysis	Method of Salivation Assessment	Potential Salivary Biomarkers
Ford et al., 1997 [24]	GD	Stimulated saliva collected over a 5 min period during which the subject chewed raw gum (chicle) and spat into a plastic container	Lysozyme activity and total protein concentration determined on fresh specimens left at 5 °C overnight; other assays performed on specimens frozen at −70 °C up to 3 weeks	Protein assay kits based on the method of Bradford, Hitachi 717 random access analyser	Flow rate (mL/min)	Up: urate (*p*-value < 0.02), potassium (*p*-value < 0.01); down: total protein, calcium (*p*-value < 0.02), lactate dehydrogenase (*p*-value < 0.01)
Higashi et al., 2011 [25]	GD	Unstimulated saliva (ca. 1 mL) directly collected into a collecting tube; at least 1 h after any food intake	Stored below −15 °C until the analysis; after thawing, centrifuged at 1000× *g* for 10 min	Stable isotope-dilution liquid chromatography/tandem mass spectrometry method	NR	T4
Morawska et al., 2021 [26]	HT	Unstimulated whole saliva collected into a centrifuge tube placed in a container with ice for 15 min, one day after blood collection and dental examination, between 7 a.m. and 9 a.m., on an empty stomach (last meal at least 10 h earlier) and did not perform any oral hygiene procedures on this day other than rinsing the mouth with water	Centrifuged for 20 min at 4 °C, 10,000× *g*, and frozen at −80 °C for no longer than 4 months, until assayed	Bio-Plex Pro Human Cytokine Assay (a multiplex assay based on magnetic beads)	Flow rate (mL/min) measured with a calibrated pipette	Cytokines: up: IL-3, IFN-γ, IL-5, IL-6, TNF-α, IL-12 (p40), HGF, IL-1α, IL-1β, IL-1RA, down: IL-8, IL-10; chemokines: up: CCL27/CTACK, CXCL1/Gro-α; growth factors: up: G-CSF, VEGF, TRIAL
Morawska et al., 2020 [27]	HT	Whole saliva unstimulated (for 15 min) and stimulated (after a 5 min break, for 5 min), collected via the spitting method into plastic centrifuge tubes placed in ice containers, between 8 a.m. and 10 a.m.; participants advised to refrain from consuming meals and drinks other than clean water, performing oral hygiene procedures for 2 h and taking any medications for 8 h; the first-minute sample discarded; stimulation triggered by dripping 100 μL 2% citric acid under the tongue every 20 s; to avoid oxidation, 0.5 M BHT added	Centrifuged for 20 min at 4 °C, 10,000× *g*, and frozen at −80 °C for no longer than 6 months, until assayed	Spectrophotometry, ELISA, colorimetry	Flow rate (mL/min) measured with a pipette calibrated to 0.1 mL	Up: total protein, IL-1β, CAT, Px, AGE, AOPP, LOOH, MDA (*p*-value < 0.0001); down: amylase, GSH, UA (*p*-value < 0.0001), SOD (*p*-value < 0.05, only unstimulated)
Pelewicz et al., 2020 [28]	GO	Using a Salivette^®^ commercial device; at three time points: directly before administration of the 1st and 12th IVMP pulses and after the cessation of oral prednisone therapy, between 8:00 and 9:00 a.m. after fasting	Stored at −20 °C until analysis	A first-generation Elecsys^®^ cortisol assay	NR	Cortisol (decreased after oral prednisone therapy)
Rao et al., 2010 [29]	HT	Unstimulated whole saliva collected by passive drooling into an ice-chilled polypropylene vial to a volume about 2 mL, at least 2 h after any food intake	Stored below −20 °C until the analyses	ELISA	NR	C-reactive protein (ns)
Tumilasci et al., 1996 [30]	GD, HT	Stimulated total parotid saliva obtained by use of a CarlsonCrittenden cannula after stimulation with a solution of citric acid 0.1 M for 3 min; between 08:00 and 09:00 a.m. after fasting	Frozen so the TRAb assay could be carried out monthly	TRAb receptor assay	Flow rate (mL/min)	TRAb
Van Herle et al., 1989 [31]	GD, HT	Unstimulated saliva from 2 to 10 mL in a clean disposable plastic container, from 5 to 30 min; patients asked to discard first salivary discharge 1 min after rinsing with water	After the collection immediately centrifugated at 10,000 rpm (5 min) and stored at −20 °C; immediately before the assays thawed and recentrifuged at 3500 rpm at 4 °C	Radioimmunoassay	NR	Tg and TgAb

Legend: AITD, autoimmune thyroid disease; GD, Graves’ disease; HT, Hashimoto’s thyroiditis; GO, Graves’ orbitopathy; NR, not reported; T4, thyroxine; IL-3, interleukin 3; IFN-γ, interferon γ; IL-5, interleukin 5; IL-6, interleukin 6; TNF-α, tumour necrosis factor α; IL-12, interleukin 12; HGF, hepatocyte growth factor; IL-1α, interleukin 1α; IL-1β, interleukin 1β; IL-1RA, interleukin 1 receptor antagonist; IL-8, interleukin 8; IL-10, interleukin 10; CCL27/CTACK, c-c motif chemokine ligand 27/cutaneous T cell-attracting chemokine; CXCL1/Gro-α G-CSF, chemokine (C-X-C motif) ligand 1/granulocyte colony stimulating factor; VEGF, vascular endothelial growth factor; BHT, butylated hydroxytoluene; ELISA, enzyme-linked immunoassay; CAT, catalase; Px, peroxidase; AGE, advanced glycation end products; AOPP, advanced oxidation protein products; LOOH, lipid hydroperoxides; MDA, malondialdehyde; GSH, reduced glutathione; UA, uric acid; SOD, superoxide dismutase; IVMP, intravenous methylprednisolone; ns, non-significant; TRAb, thyrotropin receptor antibody; Tg, thyroglobulin; TgAb, thyroglobulin antibody.

**Table 4:** Characteristics of included studies considering only quantitative assessments of saliva.

Author, Year	Setting	Study Group (F/M); Age	Control Group (F/M); Age	AITD Diagnosis	Inclusion Criteria	Exclusion Criteria	Smoking Status	Pharmacological Treatment	Method of Salivation Assessment
Agha-Hosseini et al., 2016 [32]	Iran	40 (40/0); 39.20 ± 13.8	40 (40/0); 38.95 ± 14.2	HT	Diagnosed with HT; euthyroidism at the time of the study	Smoking; neurologic drugs; steroids therapy; pregnancy; breast feeding; taking xerogenic medical agents; oral candidiasis; unfavorable oral health conditions (PPD > 3 mm); history of head and neck radiation; diagnosis of an immunological disorder, diabetes, any infectious disease, any malignancy, or any other systemic disease	Non-smokers	LT4	The flow rate calculated in milliliters per minute (under resting conditions in a quiet room between 8 a.m. and 9 a.m.); unstimulated (by expectoration without chewing movements) and stimulated (by chewing a piece of paraffin of identical size, after 60 s of pre-stimulation and swallowing the saliva present in the mouth) whole saliva collected over a period of 5 min in a calibrated and dry plastic tube
Chang et al., 2003 [33]	Taiwan	120 (67/53); 35–75	36 (13/23); 37–75	HT	History of autoimmune thyroiditis for more than 10 years from the time of diagnosis	Smoking; bad blood sugar control; autonomic neuropathy; immunorheumatic, other endocrine, gastrointestinal, hepatobiliary, renal diseases	Non-smokers	NR	Quantitative salivary scintigraphy after intravenous injection of 5 mCi Tc-99m pertechnetate (including the bilateral parotid and submandibular glands) for 30 min; at 15 min, stimulation with a 200 mg ascorbic acid tablet placed on the dorsal surface of the tongue
Changlai et al., 2002 [34]	Taiwan	40 (17/23); 41–70	Xerostomia (−): 36 (13/23); 37–75; Xerostomia (+): 25 (7/18); 35–76	HT	History of autoimmune thyroiditis for more than 10 years	Smoking; bad blood sugar control; autonomic neuropathy; immunorheumatic, other endocrine, gastrointestinal, hepatobiliary, renal diseases	Non-smokers	NR	Quantitative salivary scintigraphy after intravenous injection of 5 mCi Tc-99 m pertechnetate (including the four major salivary glands) for 30 min; at 15 min, stimulation with a 200 mg ascorbic acid tablet given orally
Coll et al., 1997 [35]	Spain	176 (152/24); 18–85 (49.3)	NA	GD, HT	Diagnosed with AITD	NR	NR	NR	Salivary gland scintigraphy
Jung et al., 2017 [36]	Republic of Korea	173 (144/29); 53.3 ± 13.3	NA	GD, HT	Symptoms of xerostomia	Drug-induced xerostomia; history of radiation therapy, surgery for head and neck tumors (including thyroid cancer)	NR	NR	Salivary gland scintigraphy after the intravenous injection of 370 MBq Tc-99 m pertechnetate (including the major salivary glands and the thyroid gland) over a 20-min period; then 10 min after the stimulation with a sialagogue the same acquisition time
Pang et al., 2021 [37]	China	32 (32/0); 36 ± 12	28 (28/0); 40 ± 12	HT accompanied by DTC.	Undergone thyroidectomy for differentiated thyroid cancer; no history of radioiodine treatment; xerostomia symptoms; hypothyroidism at the time of the study	Sjogren’ syndrome or other immunopathy; external radiotherapy to the head and neck; smoking; intake of medications that may cause xerostomia	Non-smokers	Radioiodine treatment preparation with LT4 medication (discontinued for at least one month)	Salivary gland scintigraphy after the intravenous injection of 185 MBq (5mCi) Tc-99 m pertechnetate (including the parotid glands, submandibular glands, and upper neck) over a 15-min period; at 10 min, stimulation with a 300 mg vitamin C tablet placed on the dorsal surface of the tongue
Warfvinge et al., 1992 [38]	Sweden	19 (16/3); 46–61 (54.5)	12 (8/4); 56–73 (66.8)	HT	Diagnosed with autoimmune thyroiditis	NR	NR	NR	Unstimulated whole sialometry (abnormal if ≤1.5 mL saliva in 15 min); salivary gland scintigraphy

Legend: F, females; M, males; AITD, autoimmune thyroid disease; GD, Graves’ disease; HT, Hashimoto’s thyroiditis; NR, not reported; NA, not applicable; DTC, differentiated thyroid cancer; PPD, periodontal probing depth; LT4, levothyroxine.

**Table 5:** Saliva flow rates reported in the included studies (data presented as medians with interquartile range or min–max).

Study	AITD Diagnosis	Type of Saliva	Saliva Flow Rate (mL/min)		
			Study Group	Control Group	*p*-Value
Ford et al., 1997 [24]	GD	Stimulated	1.4 (0.3–3.7)	1.2 (0.02–4.5)	<0.05
Morawska et al., 2021 [26]	HT	Unstimulated	0.27 (0.1–0.61)	0.67 (0.46–0.89)	<0.0001
Morawska et al., 2020 [27]	HT	Unstimulated	0.32 (0.07–0.77)	0.51 (0.27–0.96)	0.02
		Stimulated	0.90 (0.20–2.00)	1.01 (0.90–2.00)	ns
Agha-Hosseini et al., 2016 [32]	HT	Unstimulated	1.25 (0.90)	2.00 (2.14)	0.018
		Stimulated	2.87 (3.29)	3.50 (3.37)	0.470

Legend: AITD, autoimmune thyroid disease; GD, Graves’ disease; HT, Hashimoto’s thyroiditis; ns, not significant.

## Data Availability

Data are available on request from the corresponding author.

## References

[1] Bogusławska J., Godlewska M., Gajda E., Piekiełko-Witkowska A. (2022). Cellular and Molecular Basis of Thyroid Autoimmunity. Eur. Thyroid J..

[2] Lee H.J., Li C.W., Hammerstad S.S., Stefan M., Tomer Y. (2015). Immunogenetics of Autoimmune Thyroid Diseases: A Comprehensive Review. J. Autoimmun..

[3] McLachlan S.M., Rapoport B. (2014). Breaking Tolerance to Thyroid Antigens: Changing Concepts in Thyroid Autoimmunity. Endocr. Rev..

[4] Sawicka-Gutaj N., Gruszczyński D., Zawalna N., Nijakowski K., Muller I., Karpiński T., Salvi M., Ruchała M. (2022). Microbiota Alterations in Patients with Autoimmune Thyroid Diseases: A Systematic Review. Int. J. Mol. Sci..

[5] Bliddal S., Nielsen C.H., Feldt-Rasmussen U. (2017). Recent Advances in Understanding Autoimmune Thyroid Disease: The Tallest Tree in the Forest of Polyautoimmunity. F1000Research.

[6] Tomer Y., Huber A. (2009). The Etiology of Autoimmune Thyroid Disease: A Story of Genes and Environment. J. Autoimmun..

[7] Fröhlich E., Wahl R. (2017). Thyroid Autoimmunity: Role of Anti-Thyroid Antibodies in Thyroid and Extra-Thyroidal Diseases. Front. Immunol..

[8] Dong Y.H., Fu D.G. (2014). Autoimmune Thyroid Disease: Mechanism, Genetics and Current Knowledge. Eur. Rev. Med. Pharmacol. Sci..

[9] Ragusa F., Fallahi P., Elia G., Gonnella D., Paparo S.R., Giusti C., Churilov L.P., Ferrari S.M., Antonelli A. (2019). Hashimotos’ Thyroiditis: Epidemiology, Pathogenesis, Clinic and Therapy. Best Pract. Res. Clin. Endocrinol. Metab..

[10] Ralli M., Angeletti D., Fiore M., D’Aguanno V., Lambiase A., Artico M., de Vincentiis M., Greco A. (2020). Hashimoto’s Thyroiditis: An Update on Pathogenic Mechanisms, Diagnostic Protocols, Therapeutic Strategies, and Potential Malignant Transformation. Autoimmun. Rev..

[11] Sawicka-Gutaj N., Ziółkowska P., Wojciechowska K., Shawkat S., Czarnywojtek A., Warchoł W., Sowiński J., Szczepanek-Parulska E., Ruchała M. (2021). Eye Symptoms in Patients with Benign Thyroid Diseases. Sci. Rep..

[12] Davies T.F., Andersen S., Latif R., Nagayama Y., Barbesino G., Brito M., Eckstein A.K., Stagnaro-Green A., Kahaly G.J. (2020). Graves’ Disease. Nat. Rev. Dis. Primer.

[13] Edgar W.M. (1992). Saliva: Its Secretion, Composition and Functions. Br. Dent. J..

[14] Zhang C.-Z., Cheng X.-Q., Li J.-Y., Zhang P., Yi P., Xu X., Zhou X.-D. (2016). Saliva in the Diagnosis of Diseases. Int. J. Oral Sci..

[15] Rehak N.N., Cecco S.A., Csako G. (2000). Biochemical Composition and Electrolyte Balance of “Unstimulated” Whole Human Saliva. Clin. Chem. Lab. Med..

[16] Lee Y.-H., Wong D.T. (2009). Saliva: An Emerging Biofluid for Early Detection of Diseases. Am. J. Dent..

[17] Nijakowski K., Surdacka A. (2020). Salivary Biomarkers for Diagnosis of Inflammatory Bowel Diseases: A Systematic Review. Int. J. Mol. Sci..

[18] Maeshima E., Furukawa K., Maeshima S., Koshiba H., Sakamoto W. (2013). Hyposalivation in Autoimmune Diseases. Rheumatol. Int..

[19] Bhattarai K.R., Junjappa R., Handigund M., Kim H.-R., Chae H.-J. (2018). The Imprint of Salivary Secretion in Autoimmune Disorders and Related Pathological Conditions. Autoimmun. Rev..

[20] Kaczor-Urbanowicz K.E., Martin Carreras-Presas C., Aro K., Tu M., Garcia-Godoy F., Wong D.T. (2017). Saliva Diagnostics—Current Views and Directions. Exp. Biol. Med..

[21] Page M.J., McKenzie J.E., Bossuyt P.M., Boutron I., Hoffmann T.C., Mulrow C.D., Shamseer L., Tetzlaff J.M., Akl E.A., Brennan S.E. (2021). The PRISMA 2020 Statement: An Updated Guideline for Reporting Systematic Reviews. BMJ.

[22] NHLBI, NIH Study Quality Assessment Tools. https://www.nhlbi.nih.gov/health-topics/study-quality-assessment-tools.

[23] OCEBM Levels of Evidence. https://www.cebm.net/2016/05/ocebm-levels-of-evidence/.

[24] Ford H., Johnson L., Purdie G., Feek C. (1997). Effects of Hyperthyroidism and Radioactive Iodine given to Ablate the Thyroid on the Composition of Whole Stimulated Saliva. Clin. Endocrinol..

[25] Higashi T., Ichikawa T., Shimizu C., Nagai S., Inagaki S., Min J.Z., Chiba H., Ikegawa S., Toyo’oka T. (2011). Stable Isotope-Dilution Liquid Chromatography/Tandem Mass Spectrometry Method for Determination of Thyroxine in Saliva. J. Chromatogr. B Analyt. Technol. Biomed. Life. Sci..

[26] Morawska K., Maciejczyk M., Zięba S., Popławski Ł., Kita-Popławska A., Krętowski J., Zalewska A. (2021). Cytokine/Chemokine/Growth Factor Profiles Contribute to Understanding the Pathogenesis of the Salivary Gland Dysfunction in Euthyroid Hashimoto’s Thyroiditis Patients. Mediators Inflamm..

[27] Morawska K., Maciejczyk M., Popławski Ł., Popławska-Kita A., Kretowski A., Zalewska A. (2020). Enhanced Salivary and General Oxidative Stress in Hashimoto’s Thyroiditis Women in Euthyreosis. J. Clin. Med..

[28] Pelewicz K., Szewczyk S., Miśkiewicz P. (2020). Treatment with Intravenous Methylprednisolone in Patients with Graves’ Orbitopathy Significantly Affects Adrenal Function: Assessment of Serum, Salivary Cortisol and Serum Dehydroepiandrosterone Sulfate. J. Clin. Med..

[29] Rao N.L., Shetty S., Upadhyaya K., Prasad R.M., Lobo E.C., Kedilaya H.P., Prasad G. (2010). Salivary C-Reactive Protein in Hashimoto’s Thyroiditis and Subacute Thyroiditis. Int. J. Inflamm..

[30] Tumilasci O.R., Arqueros M.C., Ostuni M.A., el Tamer E., Houssay A.B. (1996). Thyrotropin Receptor Antibodies in Parotid Saliva. J. Endocrinol. Investig..

[31] Van Herle A.J., Rosenblit P.D., Van Herle T.L., Van Herle P., Greipel M., Kellett K. (1989). Immunoreactive Thyroglobulin in Sera and Saliva of Patients with Various Thyroid Disorders: Role of Autoantibodies. J. Endocrinol. Investig..

[32] Agha-Hosseini F., Shirzad N., Moosavi M.-S. (2016). Evaluation of Xerostomia and Salivary Flow Rate in Hashimoto’s Thyroiditis. Med. Oral Patol. Oral Cir. Bucal.

[33] Chang C.-P., Shiau Y.-C., Wang J.-J., Ho S.-T., Kao C.-H. (2003). Decreased Salivary Gland Function in Patients with Autoimmune Thyroiditis. Head Neck.

[34] Changlai S.P., Chen W.K., Chung C., Chiou S.M. (2002). Objective Evidence of Decreased Salivary Function in Patients with Autoimmune Thyroiditis (Chronic Thyroiditis, Hashimoto’s Thyroiditis). Nucl. Med. Commun..

[35] Coll J., Anglada J., Tomas S., Reth P., Goday A., Millan M., Pujol-Borrell R., Corominas J. (1997). High Prevalence of Subclinical Sjögren’s Syndrome Features in Patients with Autoimmune Thyroid Disease. J. Rheumatol..

[36] Jung J.-H., Lee C.-H., Son S.H., Jeong J.H., Jeong S.Y., Lee S.-W., Lee J., Ahn B.-C. (2017). High Prevalence of Thyroid Disease and Role of Salivary Gland Scintigraphy in Patients with Xerostomia. Nucl. Med. Mol. Imaging.

[37] Pang X.-A., Wei Z.-X., Li J.-H., Pang X.-Q. (2021). Salivary Gland Function in Women with Hashimoto’s Thyroiditis without Xerostomia and the Correlation with Auto-Thyroid Antibodies. Nukl. Nucl. Med..

[38] Warfvinge G., Larsson A., Henricsson V., Ericsson U.B., Hansen B., Manthorpe R. (1992). Salivary Gland Involvement in Autoimmune Thyroiditis, with Special Reference to the Degree of Association with Sjögren’s Syndrome. Oral Surg. Oral Med. Oral Pathol..

